# Viability of Embryo Sacs and Fruit Set in Different Plum (*Prunus domestica* L.) Cultivars Grown under Norwegian Climatic Conditions

**DOI:** 10.3390/plants11020219

**Published:** 2022-01-14

**Authors:** Radosav Cerović, Milica Fotirić Akšić, Milena Đorđević, Mekjell Meland

**Affiliations:** 1 Innovation Centre, Faculty of Technology and Metallurgy, University of Belgrade, Karnegijeva 4, 11120 Belgrade, Serbia; radosav.cerovic@gmail.com; 2 Department of Fruit Science, Faculty of Agriculture, University of Belgrade, Nemanjina 6, 11080 Belgrade, Serbia; fotiric@agrif.bg.ac.rs; 3 Department of Pomology and Fruit Breeding, Fruit Research Institute Čačak, Kralja Petra I/9, 32000 Čačak, Serbia; mdjordjevic@institut-cacak.org; 4 Department of Horticulture, NIBIO Ullensvang, Norwegian Institute of Bioeconomy Research, Ullensvangvegen 1005, N-5781 Lofthus, Norway

**Keywords:** embryo sac, fertilization, ovule, pollination, temperature

## Abstract

Compatibility and synchrony between specialized tissues of the pistil, female gametophytes and male gametophytes, are necessary for successful pollination, fertilization, and fruit set in angiosperms. The aim of the present work was to study the development and viability of embryo sacs, as well as fertilization success, in relation to the fruit set of the cultivars ‘Mallard’, ‘Edda’, ‘Jubileum’, and ‘Reeves’, under specific Norwegian climatic conditions. Emasculated, unpollinated, and open-pollinated flowers were collected at the beginning of flowering, and on the 3rd, 6th, 9th, and 12th days after flowering, from all four plum cultivars over two years (2018/2019). Ovaries were dehydrated, embedded in paraffin wax, sectioned, stained, and observed under a light microscope. Results showed the existence of synchronization between successive phases in the development of the embryo sac and individual phases of flowering. All plum cultivars had higher percentages of viable embryo sacs, fertilized embryo sacs, and fruit set in 2018 than in 2019. These differences may be related to the very low temperatures during the post-full-flowering period in 2019, and to the low adaptation of some studied cultivars to unfavorable conditions. In our study, the cultivar ‘Jubileum’ showed the highest percentage of viable embryo sacs, fertilized embryo sacs, and fruit set compared to other cultivars, i.e., the best low-temperature adaptation.

## 1. Introduction

Plum production in Norway is mainly located along the fjords in Western Norway, where winter and late-spring frosts rarely occur [1]. The long tradition of plum production in Norway started in the medieval period of Western Europe [2]. In Norway, plum ranks second among commercial fruit crops, with a harvesting area of 424.3 ha and annual production of 1524.9 tons [3]. The Gulf Stream brings heat from the tropical areas to the Norwegian Sea, where much of the warm air is emitted into the atmosphere. The most commercial plum orchards are located in Hardanger district, where the short and relatively cool growing season limits the choice of plum cultivars that can be grown [4]. Moreover, unfavorable environmental conditions during plum pollination in spring contribute to a very negative effect on fruit set and yield quantity (year-to-year variations) in plum orchards [5]. For this reason, one of the most important objectives of plum breeding in Norway is the creation or introduction of cultivars suitable for these specific climatic conditions [6].

In the plum assortment in Norway, two main self-fertile plum cultivars are predominant: ‘Opal’ and ‘Victoria’ [4]. In recent years, the cultivars ‘Edda’, ‘Mallard’, ‘Reeves’, and ‘Jubileum’ have been introduced in commercial plum orchards [7]. Previous experience indicates that the cultivars ‘Edda’, ‘Mallard’, and ‘Reeves’ are self-sterile [5], while the cultivar ‘Jubileum’ shows partial self-fertility [8].

In reproductive biology, many internal (male and female flower parts) and external factors affect fertilization success and fruit set [9]. Flowering, pollination, fertilization, and embryo development are key processes affecting fruit set rate. Processes of ovule formation and megasporogenesis, which lead to embryo sac growth, are closely related to the morphological development of the flowers. The regularity of macrosporogenesis and macrogametogenesis is also closely related to the formation of a normal and functional embryo sac [10]. Early degeneration of the embryo sac, as well as nutritional and abiotic stresses during early female development, may affect embryo abortion in some fruit species [11,12]. The viability of embryo sacs is one of the most important factors that directly controls the success of the fertilization process and, finally, fruit set [13,14].

The flower is an organ that is very sensitive to temperature fluctuations during organogenesis, before and during flowering, as well as in the post-pollination stage [15]. Temperature and humidity during flowering are the most important external factors that affect pollen viability [16], stigmatic receptivity [17], pollen tube growth in vivo [18,19], and ovule viability [20,21,22]. Embryo sac viability directly affects the effective pollination period [23]. If pollination is delayed, fertilization depends on the ovules that remain viable until the pollen tubes reach them. The receptivity of stigma and longevity of the ovule can be used to define a model based on temperature prediction [24]. The aforementioned factors and stages could play important roles in establishing optimal conditions for successful pollination and fertilization. However, unfavorable environmental conditions in spring, during the flowering period of plums, can have a very negative effect on fruit set and yield. Synchrony in male–female functionality during mating and in postzygotic stages depends more or less on the adaptability of plum cultivars to different and sometimes severe environmental conditions. Although the development of ovules is genetically determined, there are differences in the climate adaptation of cultivars to different areas [25,26]. The highest temperature can induce competition for nutrients or water between shoot growth and embryo development, thus inhibiting the phases following fertilization in some fruit species [27].

A short plum-growing season is typical in Western Norway [2]. Moreover, regardless of the applied agricultural practices, in some seasons, yields can be unsatisfactory. Fast changes in temperature, frequent rainfall, low sunlight, and strong winds can occur during the flowering period of plums. Previously, the progamic phase of fertilization and fruit set in the cultivars ‘Mallard’, ‘Edda’, ‘Jubileum’, and ‘Reeves’ was observed, in an attempt to determine the best pollinizers for those cultivars [14]. Furthermore, in these plum cultivars, pollen donors and success rates of individual pollinizers using microsatellite markers have been identified [1], together with the determination of the effective pollination period under the specific Nordic climate [28].

In this study, the most important aspects of the functionality of the embryo sac—such as embryo sac development and viability in relation to fertilization success and fruit set of four plum cultivars (‘Mallard’, ‘Edda’, ‘Jubileum’, and ‘Reeves’) in open pollination—were analyzed. The aim was to provide complete knowledge of the reproductive biology of these plum cultivars, and to make it possible to choose the best cultivar in order to ensure high fruit set and stable yields.

## 2. Results

### 2.1. Time of Flowering and Climatic Conditions

**Figure 1:**
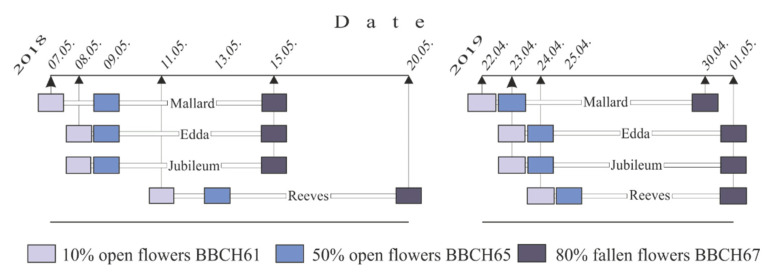
Flowering phenophases of plum cultivars in 2018 and 2019.

During 2018, ‘Mallard’ had the earliest beginning of flowering (7 May) and 9-day flowering interval (7–15 May) (Figure 1). The flowering period of the cultivars ‘Edda’ and ‘Jubileum’ was one day shorter (8–15 May). ‘Reeves’ had the latest and longest interval of flowering (11–20 May). The following year, the beginning of flowering (BOF) started 15–17 days earlier in all cultivars. In 2019, the order of the beginning of flowering of the studied cultivars was the same as in the previous year. In this year, ‘Mallard’ was the first to start flowering (22 April), two days earlier than the latest cultivar ‘Reeves’ (24 April). The flowering interval, depending on the cultivar, was slightly shorter in 2019 (8–9 days) compared to 2018 (8–10 days). In both years, full flowering started on the second day of BOF, except for ‘Mallard’ and ‘Reeves’, whose flowering began on the third day in 2018.

During the plum-growing season, the average daily temperature in 2018 was 13.6 °C, and in the following year it was 12.9 °C. However, climatic conditions in this part of Norway influenced the different beginnings of flowering in these two years. In April 2019, the average temperature was 9.1 °C, while it was 6.3 °C in April 2018. In 2019, higher temperatures at the beginning of spring caused the start of plum flowering almost two weeks earlier than usual for this part of Norway. The mean daily temperature during the flowering period of all cultivars was 14.3 °C in 2018; during this period, the mean maximum temperature was 20.1 °C, while the mean minimum temperature was 9.6 °C. In 2019, daily temperature during the flowering period was slightly lower, at 14.0 °C; in this year, the mean maximum temperature was 18.8 °C, while the mean minimum temperature was 10.3 °C. The mean daily precipitation was 0.7 mm in 2018 and 0.5 mm in 2019.

On the other hand, the average temperature in May 2018 was 4.9 °C higher than for the same period in 2019, giving good conditions for fruit set and early fruitlet development. In 2018, the average daily temperature during the 10-day post-flowering period was 18.1 °C; during this year, average maximum temperature was 24.4 °C, while the average minimum temperature was 12.6 °C. In the following year, the average temperature was only 5.9 °C, with an average maximum temperature of 9.4 °C and an average minimum temperature of 2.9 °C.

### 2.2. Development and Viability of Embryo Sacs

**Figure 2:**
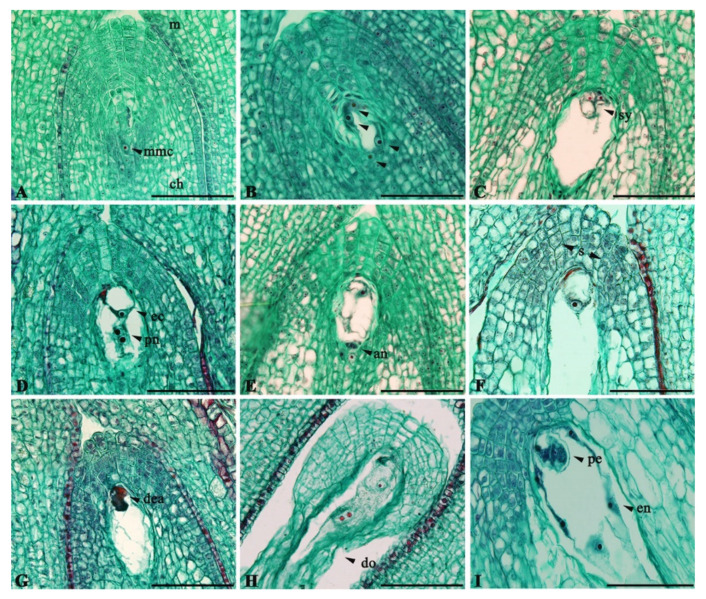
Embryo sac development in plum cultivars: (**A**) Megaspore mother cell—‘Edda’. (**B**) Four-nucleate state after second mitotic division—‘Mallard’. (**C**) Position of synergid cells—‘Jubileum’. (**D**) Normal position of the egg cell and polar nuclei—‘Jubileum’. (**E**) Antipodal cells—‘Reeves’. (**F**) Starch grains in nucellar cap tissue—‘Jubileum’. (**G**) Degeneration of elements in the embryo sac—‘Reeves’. (**H**) Degeneration of the ovule—‘Mallard’. (**I**) Early embryogenesis—‘Edda’. m: micropylar pole; ch: chalazal pole; mmc: megaspore mother cell; arrowheads: nuclei; s: synergid cells; ec: egg cell; pn: polar nuclei; an: antipodal cells; s: starch grains; dea: degenerated egg apparatus; do: degenerated ovule; pe: pro-embryo; en: endosperm nuclei. Scale bars = 200 μm.

All analyzed plum ovaries consisted of one locule, with a common axis from which two ovules were attached. The ovules were characterized by anatropic position, bitegmic and crassinucellate integuments, and short funiculus. These ovules were approximately the same size up to the balloon stage of the flower. At anthesis, the histological structure and shape of the ovules changed rapidly; one ovule quickly atrophied, while another one developed into seeds if fertilized. In these studies, only the larger ovules that remained functional after the balloon phase were analyzed. The process of megasporogenesis was not studied in this experiment, since its development starts earlier, so the mother megaspore cell (or process of megasporogenesis) is rarely seen at the beginning of flowering (Figure 2A). Sequences of the process of megagametogenesis (three mitotic cycles) were seen in some plum cultivars at the beginning of flowering (Figure 2B). This process ended with the formation of monosporous bipolar embryo sacs with eight nuclei.

During full flowering, the mature embryo sac contained two synergids, one egg cell, and a central cell with two polar nuclei and three antipodes. The egg apparatus with two side-by-side pear-shaped synergids with large vacuoles and nuclei in their upper parts was located in the upper part of the embryo sac (Figure 2C). The egg cell was located between the upper parts of the synergids, with a large vacuole and nucleus located in a sickle zone of the egg cytoplasm (Figure 2D). The central cell contained two polar nuclei located closer to the egg apparatus (Figure 2D). Three ephemeral antipodal cells were situated at the chalazal pole of the embryo sac (Figure 2E); the antipodes decayed rapidly. In the unpollinated conditions, embryo sacs with two polar nuclei were normally observed in the middle stages of full flowering, i.e., embryo sacs with five nuclei. At a later stage of full flowering, fusion of the polar nuclei into a single central nucleus took place. Before syngamy, it is common for the embryo sac to have four nuclei; at this stage, the typical elongation of the embryo sac towards the chalazal part of the ovule was observed. On the sixth day after BOF, different concentrations of starch grains were observed in the cytoplasm, around the nucleus of the central cell, and in the tissue of the ovule’s nucellar cap (Figure 2F). In both years, after full flowering, typical phenomena of irregularities were observed in the spatial position and cytological shape of individual elements of the embryo sac, along with partially or fully degenerated embryo sacs (Figure 2G). The central nucleus showed the longest viability in the embryo sac. In parallel with the described irregular arrangement of individual elements and degenerative changes in embryo sacs, the embryonic tissue also showed signs of degeneration. Usually, such phenomena were observed first in the integuments, spreading later to the whole ovule (Figure 2H). These phenomena were more pronounced after the sixth day post-BOF. In open pollination, in the later phase of full flowering, we recorded the occurrence of early stages of embryo (pro-embryo or globular embryo) and the process of endosperm formation (Figure 2I). In 2018, these phenomena were observed in ‘Edda’, ‘Jubileum’, and ‘Reeves’ on the sixth day after BOF, while in the following year they were recorded on the ninth day after BOF in all cultivars.

**Figure 3:**
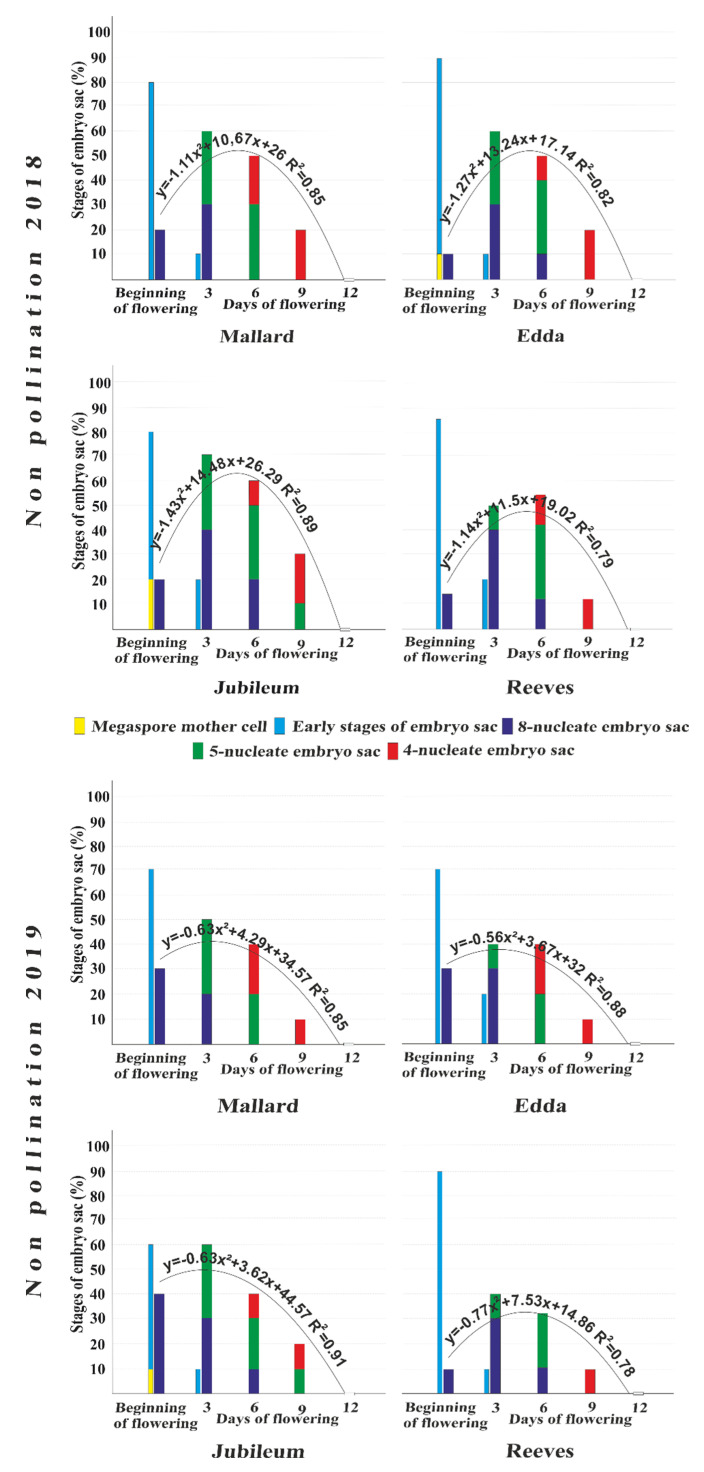
Regression analysis of embryo sac viability (%) in unpollinated flowers at the BOF, and on the 3rd, 6th, 9th, and 12th days after BOF in the plum cultivars ‘Mallard’, ‘Edda’, ‘Jubileum’, and ‘Reeves’ during 2018 and 2019.

The dynamic of embryo sac development, from the beginning of flowering until the 12th day after BOF in unpollinated flowers, is shown in Figure 3.

Embryo sacs with megaspore mother cells were recorded at the beginning of full flowering in ‘Edda’ in 2018, and in ‘Jubileum’ in both years. The percentage of embryo sacs with megaspore mother cells was between 10 and 20%. In this phase of flowering beyond the early stages of embryo sac development, fully developed eight-nucleate embryo sacs were present in all cultivars in both years. This embryo sac stage was maintained up to the sixth day after BOF in ‘Jubileum’ and ‘Reeves’ in both years, as well as in ‘Edda’ in 2018. The embryo sacs with five nuclei were recorded on the third and sixth days after BOF in all plum cultivars in both years; this stage of the embryo sac was also present on the ninth day after BOF, but only in ‘Jubileum’. Depending on the cultivar, the percentage of embryo sacs at this stage was between 10 and 30%. In both years, the last functional stage (four-nucleate embryo sac) was observed on the sixth and ninth days after BOF, except in ‘Reeves’, where it appeared only on the ninth day after BOF in 2019. The percentage of these stages in studied plum cultivars did not exceed 20%. On the 12th day after BOF, no functional stages of embryo sacs (eight-nucleate, five-nucleate, or four-nucleate) were observed in any of the tested cultivars. The cultivar ‘Jubileum’ had the highest percentage of functional sacs (in total) on the third and sixth days after BOF in both years. The average number of embryo sacs from these two terms was 65% in 2018 and 50% in 2019. The lowest percentage of functional embryo sacs was found in the cultivar ‘Reeves’ in both years; the average percentage in this cultivar (total for all terms) was 26.4% in 2018 and 18.7% in 2019.

In unpollinated flowers, all three functional stages of the embryo sac that are capable of fertilization (eight-, five- and four-nucleate) were presented cumulatively with a regression line of the parabola type, presenting a trend of embryo sac viability during flowering. The values of the multiple correlation coefficients (R), ranging from 0.78 to 0.91, showed that these studied stages were highly correlated with one another in both years for all studied cultivars. The regression trend of cumulative viability of embryo sacs showed higher values in 2018 than in 2019 for all studied cultivars (Figure 3). The regression trend in 2019 shows that the high initial percentage of functional embryo sacs at the beginning of full bloom does not mean a higher percentage of functional and longer viable embryo sacs in the later stages of full flowering. Thus, in 2019, the average percentage of eight-nucleate embryo sacs within all tested cultivars was 27.5%, while in 2018 it was only 16.1%. However, the cumulative percentage of functional embryo sacs was generally lower in terms of flowering in 2019 than in 2018. On the other hand, regression trends showed a faster decline in the percentage of viable embryo sacs in 2018 compared to 2019. The highest trend of functional stages (eight-, five-, and four-nucleate embryo sacs) was seen in the cultivar ‘Jubileum’ in 2018, while the lowest was in the cultivar ‘Reeves’ in 2019. A decreasing trend of embryo sac functionality was observed between the sixth and the ninth days after BOF in both years, and for all cultivars tested. The highest percentage of functional embryo sacs was recorded in the cultivar ‘Jubileum’ on the ninth day after BOF in both years (30% in 2018 and 20% in 2019). A lower percentage of functional embryo sacs (10%) in the same period of full flowering was observed in ‘Mallard‘, ‘Edda’, and ‘Reeves’. On the 12th day after the beginning of full flowering, there were no vital functional embryo sacs (eight-, five-, or four-nucleate). The embryo sacs showed a slightly faster trend of decline in their viability in 2018 than 2019 in the period from the 9th to the 12th days after BOF.

### 2.3. Fertilization Success and Fruit Set

**Figure 4:**
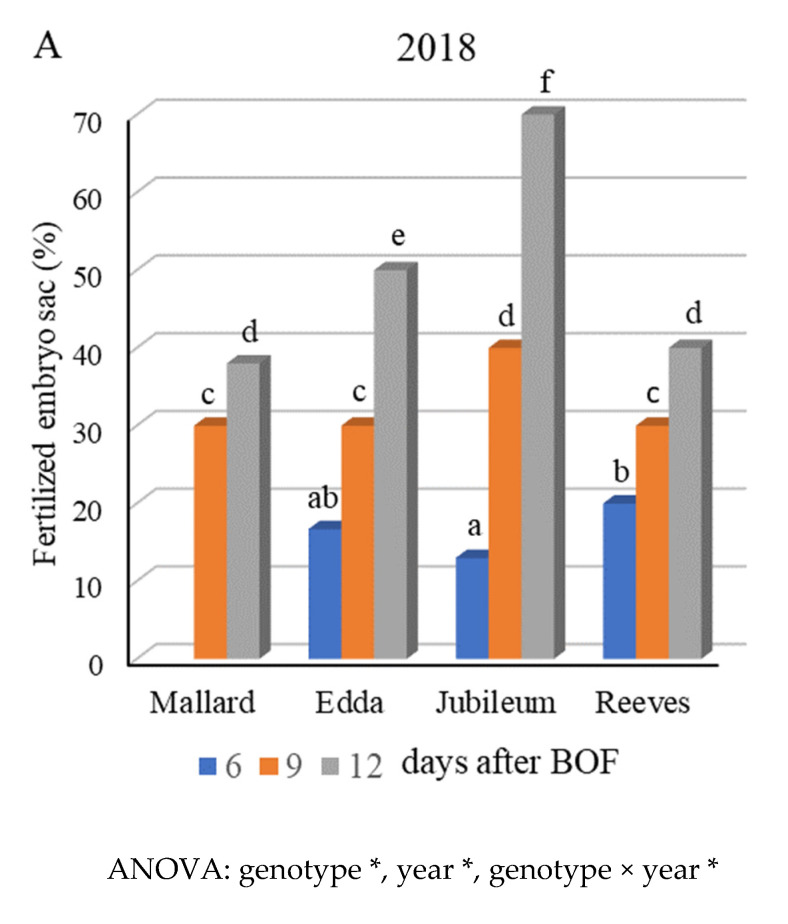

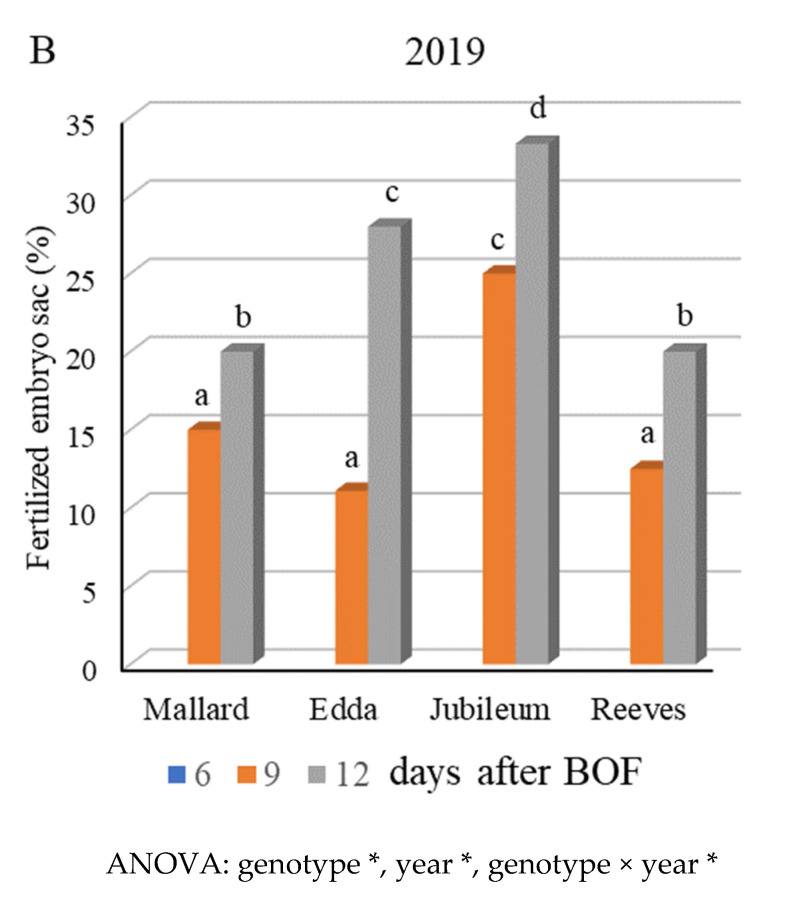
Percentage of fertilized embryo sacs in plum cultivars ‘Mallard’, ‘Edda’, ‘Jubileum’, and ‘Reeves’ in open-pollination conditions, during 2018 (**A**) and 2019 (**B**). (Different letters above the bars denote a significant difference between cultivars according to the LSD test, *p* < 0.05; asterisk (*) very significant differences).

Fertilization success, which is directly related to the percentage of fertilized embryo sacs in plum cultivars, was examined during flowering (from the beginning of flowering until the 12th day after BOF) under conditions of open pollination (Figure 4). Fertilized egg cells and different early embryo structures were observed in accordance with ontogenetic development (pro-embryo or globular stage). It is common for synergids to degenerate before the pollen tube arrives. At this stage, the embryo sac has four nuclei. In some cases, it was recorded that pollen tubes with male gametes entered the embryo sac between the two synergids without destroying either of them.

In 2018, fertilized embryo sacs were observed in all cultivars (except for ‘Mallard‘) on the sixth day after BOF—three days earlier than in 2019 (9 days post-BOF). The cultivar ‘Jubileum’ had the highest percentage of fertilized embryo sacs (70%) on the 12th day after BOF. In this year, the lowest percentage of fertilized embryo sacs was recorded in ‘Mallard‘ (38%). The following year, the percentage of fertilized embryo sacs was much lower. The cultivar ‘Jubileum’ had the highest (33.3%), followed by ‘Edda’ (28%), and then by ‘Mallard‘ and ‘Reeves’ (20% in both). ANOVA showed the statistical significance for all factors and interactions (results not shown).

**Figure 5:**
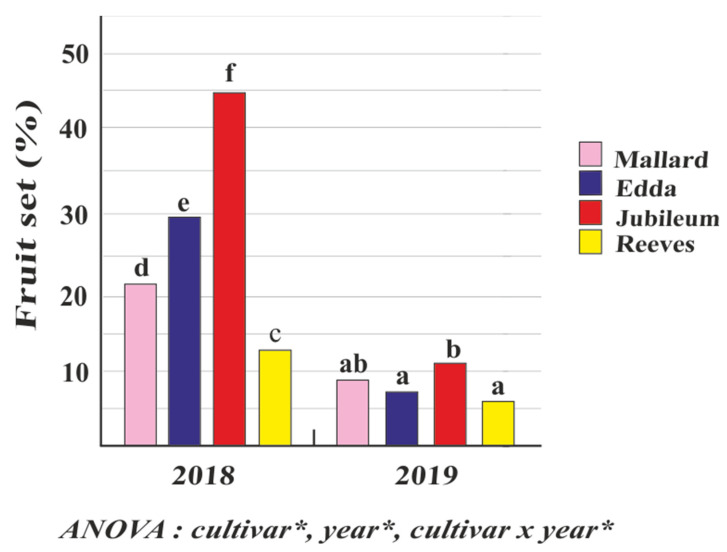
Fruit set in the plum cultivars ‘Mallard’, ‘Edda’, ‘Jubileum’, and ‘Reeves’ during 2018 and 2019 in open-pollination conditions. (Different letters above the bars denote a significant difference between cultivars according to the LSD test, *p* < 0.05; asterisk (*) very significant differences).

The fruit set values during open pollination are given in Figure 5. The percentages of fruit set in all cultivars were higher in 2018 than in 2019. Taking into account the cumulative values for all cultivars, the average percentage of fruit set was 27.3% in 2018 and 8.2% in 2019 (3.3-fold less). The highest percentage of fruit set in 2018 and in 2019 was recorded in ‘Jubileum’ (44.7% and 10.9%, respectively). In both years, ‘Reeves’ had the lowest percentage of fruit set (13% in 2018 and 5.4% in 2019). In 2018, ‘Edda’ (29.7%) had slightly better results than ‘Mallard‘ (21.6%); in contrast, in 2019, ‘Mallard‘ (9%) had higher fruit set than ‘Edda’ (7.4%).

## 3. Discussion

### 3.1. Development and Viability of Embryo Sacs

Two ovules per ovary are typical of the genus *Prunus*, as has been confirmed in many fruit species, such as almonds [25,29], apricots [30,31], peaches [32], cherries [12,33], Japanese plums [26,34], and European plums [35,36]. Moreover, the presence of two ovules per ovary was confirmed in all examined cultivars. These ovules were approximately the same size at the beginning of flowering. From the anthesis, one of the ovules can be recognized as the primary ovule which, if fertilized, develops a seed—unlike the secondary ovule, which atrophies. The different starch reserves inside both ovules determine their fate [37,38]. The starch level in both ovules is the same, but shortly after anthesis the starch reserves are withdrawn from one ovule [39].

Plum embryo sac belongs to the monosporous eight-nucleate bipolar *Polygonum* type [40]. Generally, female gametophytes play an important role in the reproductive process, including pollen tube guidance, fertilization, and seed development upon fertilization [41,42]. Huang and Russel [43] proved that synergids play a key role in directing the growth of the pollen tube towards the egg apparatus. Furthermore, evidence of starch grains in the nucellar cup cells also suggests a direct flow of starch grains along the embryo sac [44]. Skinner and Sundaresan [45] demonstrated that cell-to-cell communication within the gametophyte is crucial for maintaining cell identity, as well as for facilitating double fertilization. This is especially important, because the interval between the discharge of male gametes from the pollen tubes and their fusion with egg cells is very short.

At the beginning of the flowering, the embryo sac is cellularized, and consists of an egg apparatus (two synergids and an egg cell) and a central cell with two polar nuclei and three antipodal cells in all cultivars (eight-nucleate embryo sac). In both years, functional embryo sacs with five nuclei and four nuclei were recorded from the third to the ninth days after BOF in all studied plum cultivars. In this experiment, the antipodes were present for up to six days after BOF in most of the cultivars. The function of antipodal cells is not clear, but their degeneration occurs prior to fertilization in many flowering plants [46]. In all cultivars, the polar nuclei fuse with one another, forming a central nucleus (2n), before they are fertilized by a single sperm nucleus (n) and form an endosperm (3n) [44]. In our results, the central nucleus showed the longest viability; despite the atrophy of the entire ovule, it was still vital.

The order of successive stages of embryo sac development (eight nuclei, five nuclei, and four nuclei) showed the usual dynamics during the flowering period in all cultivars, as has been previously reported for sour cherry [10] and plum [19]. However, in other fruit species, flowers at the same external stage of development showed heterogeneity of ovule (megagametophyte) progression stages [13,47]. The high temperatures before anthesis can affect the lack of synchronization between the external phenological stage and ovule development [27,48]. In our work, the existence of synchronization between successive phases in the development of the embryo sac and individual phases of flowering was determined. In both years, the studied plum cultivars showed differences only in the number of functional stages during the flowering period, regardless of the significant time difference in terms of the beginning of flowering (beginning of May in 2018 and end of April in 2019). These consistent differences between cultivars during both years indicated a genetic determination [26]. On the other hand, slow development of the embryo sacs can lead to a high number of ovules with undifferentiated embryo sacs [13]. A certain degree of megagametophyte development at full bloom may not be enough to ensure a high rate of fruit set. In some cases, high percentages of functional ovules had low fruit set [31].

The viability of embryo sacs is an important factor in the reproductive process, directly affecting successful fertilization and fruit set. In both years, the trend of embryo sac viability in all plum cultivars showed direct correlation with the number of functional embryo sacs. In 2018, the percentage of functional embryo sacs in all cultivars was higher compared to 2019. In both years, the cultivar ‘Jubileum’ had the highest percentage of viable embryo sacs (36% in 2018 and 32% in 2019), while ‘Reeves’ had the lowest (26.4% in 2018 and 18.7% in 2019), throughout the whole period of flowering. The average daily temperatures during the flowering period were similar—14.3 °C in 2018, and 14 °C in 2019. [26]. The loss of embryo sac viability was recorded on the third day after BOF in all cultivars except for ‘Reeves’ in 2018 and ‘Edda’ in 2019, where the senescence started on the sixth day after BOF. The progressive degeneration of embryo sacs began on the 6th day after BOF, while non-viable embryo sacs were found on the 12th day after BOF. The differences in the decrease in ovule viability were genotype-dependent, as previously determined by Cerović et al. [20] and Ruiz et al. [26]. The degeneration of the embryo sac followed the occurrence of irregular spatial distribution of the egg apparatus, degeneration of elements in the embryo sac, degeneration of the whole embryo sac and, finally, ovule atrophy, as described by Cerović and Mićić [10] and Đorđević et al. [49].

### 3.2. Fertilization Success and Fruit Set

Successful fertilization occurs in those cases where male and female gametes are in the same phase of the cell cycle [50]. In some stone fruits, the viability of ovules is particularly short [51]. Higher temperatures accelerate the loss of ovule viability and, thus, reduce the success of fertilization [52]. According to Stösser and Anvary [53], plums lose ovule viability in 3–4 days at a temperature of 20 °C. In addition to higher temperatures, Italian plum cultivars can lose of embryo sac viability at lower temperatures [35]. In some plum cultivars, low temperature can slow the growth of pollen tubes so much that they cannot reach the functional ovule [54]. The viability of embryo sacs, as well as the growth rate of pollen tubes in vivo in individual pollinizers, represents the genotypic specificity of certain cultivars of pome and stone fruits in relation to certain temperatures [9].

Our results show that the percentage of viable and fertilized embryo sacs and embryos was higher in all cultivars in 2018 compared to 2019. The average number of viable embryo sacs for the entire examined period, in the first and second years, was 30.1% and 25.2%, respectively. The average number of fertilized embryo sacs in 2018 (33.1%) was ~2.8-fold higher than in 2019 (11.8%). Similar results were obtained for fruit set, where cultivars in 2018 (27.3%) had ~3.3-fold higher fruit set than in 2019 (8.2%). The appearance of fertilized egg cells, early embryo structures, and nucleate endosperm in fertilized embryo sacs was observed on the sixth day after BOF in 2018 and the ninth day after BOF in 2019. Similar results were also observed in apricot [55], sour cherry [10], and pear [56]. Among the studied cultivars in both years, the best percentage of fertilized embryo sacs and fruit set was recorded in the cultivar ‘Jubileum’.

In our study, values of fertilized embryo sacs had a direct influence on fruit set. Moreover, percentages of fertilized embryo sac and fruit set showed the close relationship in all cultivars during periods of flowering where average daily temperatures were similar. Temperature is one of the key limiting factors during sequences of the reproductive process [57,58]. Temperature conditions in the post-full-flowering period could have caused the higher percentage of fertilized embryo sacs and fruit set in 2018 compared to 2019. In 2018, the average temperature was 4.9 °C higher than in 2019. The previous study, which evaluated pollinizers and fertilization of the same plum cultivars, showed higher fruit set in 2018 [14]. In this study, higher values of fruit set were obtained in both years, as a consequence of artificial pollination. However, according to Fotirić Akšić et al. [28], regardless of the differences in climatic conditions in those two seasons, EPP was 3 days for ‘Mallard’, ‘Edda’, and ‘Reeves’ in both years, and 5 days for ‘Jubileum’ in both years. In our study, under open pollination, the values of fertilized embryo sacs and fruit set in the studied plum cultivars showed temperature dependence—especially in the post-full-flowering period.

## 4. Materials and Methods

### 4.1. Plant Material and Experimental Design

The plum cultivars ‘Mallard’ (old English plum seedling), ‘Edda’ (‘Czar’ × ‘Pêche’), ‘Jubileum’ (‘Giant’ × ‘Yakima’), and ‘Reeves’ (‘Prune Peche’ × open pollinated, Canadian plum seedling) were used for the study of embryo sac viability and fruit set in the two years (2018/2019). The study was carried out in an experimental plum orchard in Leikanger (61°10′43.2′′ N; 6°51′34.3′′ E) at the Njøs Fruit and Berry Center, Western Norway. The plum orchard was established in 2012. All cultivars were grafted on the rootstock ‘St. Julien A’. The orientation of the rows of trees was east–west. The trees were trained as slender spindle trees with a maximum of 2.5 m height. Grass in the inter-rows and a 1 m wide vegetation free strip in the intra-row space were used for orchard floor management. The trees were irrigated when water deficits occurred.

Five trees with uniform branches and uniformity of flowering of each cultivar were used for this experiment. One hundred flowers per cultivar were emasculated at the late balloon stage and left unpollinated for the study of embryo sac functionality (development and viability). Emasculated flowers were not isolated with bags, so as to avoid damage to pistils in case of unfavorable weather conditions such as rain and wind. Another group of 100 flowers per cultivar were counted and left for open pollination in order to study fertilization success. In these experiments, a total of 800 flowers were used. Various pollinators were present in the orchard during these experiments.

### 4.2. Flowering Time and Climate Conditions

The Biologische Bundesanstalt, Bundessortenamt, und Chemische Industrie (BBCH) scale was used for determination of the different phenophases of flowering [59]. In accordance with the BBCH scale, the date of the beginning of flowering (BBCH stage 61) was determined when approximately 10% of flowers were open, full flowering (BBCH stage 65) was recorded when 50% of flowers were open, while the end of flowering was noted when the majority of petals were fallen (BBCH stage 67).

Western Norway has a marine climate, with comparatively cool summers and mild winters. Weather fronts usually come from the Atlantic Ocean, so clouds, rain, and wind dominate throughout the year. An average annual air temperature of 6.6 °C and annual rainfall of 994 mm are typical for the fjord areas of Western Norway. Unfavorable environmental conditions—especially low temperatures and rain—can often occur during spring. The annual average temperature in both years of study (2018/2019) was the same, at 8.1 °C. The lowest temperature was recorded on 1 March (−11.9 °C), and the highest on 28 July (31.6 °C), in 2018. The following year, the temperature also varied, where the lowest temperature was recorded on 6 February (−6.6 °C) and the highest on 28 July (32.5 °C). The rainy days were more numerous in 2019 (133) than in 2018 (115). Unfavorable environmental effects—especially low temperatures and high deposition—can often occur during March–May. In both years the total amount of annual precipitation was similar—1024 mm in 2018, and 1033 mm in 2019. However, the amount of precipitation was quite different in March, April, and May. During these months, key processes take place, such as swelling of buds, flowering and leafing, fertilization, and fruit set. During this period in 2018, the total amount of rainfall was 82.5 mm, while in 2019 it was 171 mm; the average temperatures for same period were 7.1 °C in 2018 and 7.3 °C in 2019.

In this study, minimal, maximal, and average air temperatures together with the daily amounts of rainfall (mm) were recorded during the flowering of these plum cultivars.

### 4.3. Microscopic Preparations

Flowers from both groups (for the study of development and viability, as well as fertilization success) were taken at the beginning of flowering, and on the 3rd, 6th, 9th, and 12th days after the beginning of flowering (BOF). The ovaries were fixed in FPA (formalin: propionic acid: 70% ethyl alcohol, 5:5:90) and stored at 4 °C. The material was dehydrated through a series of ethyl alcohol and then molded into paraffin blocks, which were then cut longitudinally at 10 µm by a Leica RM 2155 (Leica Microsystems Nussloch GmbH, D-69226 Nussloch; Germany) rotary microtome. Slices that were mounted on and adhered to glass slides were stained with safranin, crystal violet, and light green SF, as described by Gerlach [60]. Permanent stained slices were observed and photographed under an Olympus BX61 microscope (Tokyo, Japan).

### 4.4. Fruit Set

For determination of fruit set in open-pollination conditions, a group of 250–300 flowers were chosen for each plum cultivar (number of fruits/total number of flowers × 100). The percentage of fruit set was counted just before harvest.

### 4.5. Statistical Analysis

The application of regression lines allowed us to determine the best fitted trend between functional stages of embryo sacs with regard to their viability and days from BOF until the 12th day after.

The data obtained for fertilized embryo sacs and fruit set were statistically analyzed using Fisher’s model of two-factor analyses of variance (ANOVA). The significance of the individual differences for the investigated factors (cultivar, year, and their interactions) was determined using the least significant difference (LSD 0.05 = 95% confidence). Statistical analyses were conducted using STATISTICA for Windows 6.0 (StatSoft Inc., Tulsa, OK, USA).

## 5. Conclusions

The functionality of embryo sacs, fertilization success, and fruit set were studied for two years in the plum cultivars ‘Mallard’, ‘Edda’, ‘Jubileum’, and ‘Reeves’, under the climatic conditions of Western Norway. The greater variation in temperature conditions had a negative effect on the yield performance of these cultivars. In addition to the existence of other important reproductive factors, it is necessary to improve the knowledge about the impact and role of the female factors in the fruit set of these plum cultivars. The genomic plasticity of these plum cultivars and their adaptability to unfavorable climatic conditions is crucial for their successful cultivation.

In this study, synchronization between successive phases in the development of the embryo sac and individual phases of flowering existed in both years. This especially refers to the number of functional stages and trend of viability of embryo sacs, as well as fertilized embryo sacs and fruit set. All plum cultivars had a higher percentage of viable, fertilized embryo sacs and fruit set in 2018 than in 2019. These differences can be attributed to the very low temperatures during the post-full-flowering period in 2019. This indicates reduced adaptation to low temperatures in some of the studied plum cultivars. The cultivar ‘Jubileum’ showed the best percentages in all parameters studied herein—such as percentages of viable embryo sacs, fertilized embryo sacs, and fruit set—compared to other cultivars, i.e., best low-temperature adaptation.

By knowing which temperature regime is optimal for growing plums, planting of orchards and the prediction of high fertilization success and fruit set in the studied plum cultivars could be improved. In practical terms, this means that test prediction models of embryo sac functionality concerning the impact of environmental factors (temperature) on plum production in Western Norway can be formulated.

## Data Availability

All data are presented in this manuscript.
